# Endoscopic Characteristics of Small-Bowel Gastrointestinal Stromal Tumors Detected During Single-Balloon Enteroscopy: A Retrospective Analysis

**DOI:** 10.5152/tjg.2024.23156

**Published:** 2024-05-01

**Authors:** Xueyong Zuo, Zhong Guan, Yun Zhuang, Jianping Chen, Mei Wang

**Affiliations:** 1 Department of Gastroenterology, The First People’s Hospital of Changzhou, The Third Affiliated Hospital of Soochow University, Changzhou, Jiangsu, China; 2 Department of Gastrointestinal Surgery, The First People’s Hospital of Changzhou, The Third Affiliated Hospital of Soochow University, Changzhou, Jiangsu, China

**Keywords:** Enteroscopy, gastrointestinal stromal tumors, overt gastrointestinal bleeding, single-balloon enteroscopy

## Abstract

**Background/Aims:**

The endoscopic features of small-bowel gastrointestinal stromal tumors (GISTs) are not well defined. The objective of this study was to describe the endoscopic features of GISTs of the small intestine detected via single-balloon enteroscopy (SBE).

**Materials and Methods:**

Patients with surgically confirmed small intestinal GISTs from January 2014 to September 2022 were retrospectively analyzed. The hospital’s electronic medical record system was used to retrieve the patients’ data, including their demographics, clinical symptoms, hemoglobin on admission, endoscopic and computerized tomography findings, clinicopathological findings, and surgical management data.

**Results:**

In total, 46 GIST patients (23 men and 23 women) with overt bleeding were included, with a mean age of 52 years (23-80 years). The typical duration of the symptoms was 48 hours. Four patients (8.70%) had lesions in the duodenum, 32 (69.56%) had lesions in the jejunum, 8 (17.39%) had lesions in the ileum, and 2 (4.35%) had lesions around the junction of the jejunum and ileum. Out of the 46 patients, 27 underwent SBE, and GISTs were visualized in 25, while the lesions could not be visualized in the remaining 2. Submucosal round (n = 13), submucosal sessile (n = 8), and invasive/penetrating (n = 4) were among the endoscopic tumor features. Twenty patients exhibited submucosal protuberant lesions, with ulceration, vascular nodules/congestion, or erosion on the surface, and 5 patients presented ulcerative infiltrative lesions. The multiple logistic regression analysis indicated that the invasive/penetrating characteristics of GISTs under SBE evaluation are significantly correlated with the risk level of GIST malignancy (*P* < .05).

**Conclusion:**

A variety of endoscopic characteristics could be observed during the preoperative SBE evaluation of small-intestine GISTs.

**Main Points**

- Among the 25 gastrointestinal stromal tumor (GIST) patients, 15 (60%) had submucosal round lesions, while 8 (32%) had lesions with a submucosal sessile appearance and 5 (20%) with an invasive/penetrating appearance on single-balloon enteroscopy (SBE).
- The invasive/penetrating characteristics of GIST under SBE evaluation are significantly correlated with the risk level of GIST malignancy.
- There is a lack of information on the endoscopic features of small intestine GISTs, and this work contributes novel information to the body of knowledge.

## Introduction

Approximately 0.1%-3% of all gastrointestinal malignancies are gastrointestinal stromal tumors (GISTs), which are rare mesenchymal tumors in the wall of the gastrointestinal tract.^1^ This type of tumor can develop anywhere in the gastrointestinal system, occasionally even outside of it, with the stomach hosting 60% and the small intestine 30% of all GISTs.^2^ Small-intestine GISTs are associated with a poorer prognosis compared with gastric GISTs, a purportedly higher prevalence, and more aggressive biobehavioral characteristics.^3^ Therefore, an early accurate diagnosis should be mandatory to improve the survival of patients with small intestinal GISTs. However, due to their relative rarity and vague clinical signs, this type of GIST frequently presents a diagnostic challenge.^4^

As the middle part of the gastrointestinal tract, the small intestine is tortuous and has a large range of movement, making it even more difficult to diagnose diseases in this segment.^5^ The whole small bowel can now be observed due to the technological advancements in endoscopic techniques, including video capsule endoscopy, single-balloon enteroscopy (SBE), and double-balloon enteroscopy (DBE).^6^ Yoo et al’s^4^ description of the clinicopathological characteristics of small-intestine GISTs identified via DBE was lacking a description of the variety of endoscopic presentations of these tumors. According to Dolu et al,^7^ small-intestine tumors can be categorized as polyps or masses based on the endoscopic presentations and as benign or malignant based on the histological characteristics. However, there are few descriptions of the endoscopic features of small-bowel GISTs. The diagnosis and treatment of small-bowel illnesses may be accomplished safely and effectively using SBE.^8^ Nonetheless, how SBE is used in small-intestine GISTs and the attendant clinicopathologic characteristics have not been documented. This research is thus an observational study involving GIST patients. The aim of this study is to determine the clinical use of SBE in the diagnosis of small-bowel GISTs and to define the attendant endoscopic features.

## Materials and Methods

### Patient Selection

Retrospective analysis was conducted on patients hospitalized in the gastroenterology and gastrointestinal surgery departments of The First People’s Hospital of Changzhou from January 2014 to September 2022 who had pathologically confirmed small-intestine GISTs following surgery. The inclusion criteria were as follows: (1) overt gastrointestinal bleeding was present; (2) patients with a full medical history; (3) patients with strong cooperation; (4) patients with normal verbal communication abilities; (5) pathological confirmation via laparotomy or laparoscopic surgery; and (6) the absence of any further cancers. The exclusion criteria were as follows: (1) patients under 18 years old; (2) pregnancy; (3) patients unable to tolerate surgery or refusing surgery; (4) patients with contraindications to the use of anesthesia or surgery, such as cardiopulmonary insufficiency; (5) patients with an incomplete medical history; (6) patients with no compliance; (7) patients with no verbal communication skills; and (8) patients with an associated malignancy.

The study was conducted in accordance with the Helsinki Declaration and was approved by the Ethics Committee of The First People’s Hospital of Changzhou (Approval number: 2023024, Date: 2023). Signed, informed consent was obtained from the participants/legal guardians included in this study.

### Data Collection

To gather information on the patients, the hospital’s electronic medical record system was accessed for the relevant data, including in terms of demographics, clinical symptoms, hemoglobin levels on admission, endoscopic and computerized tomography (CT) findings, clinicopathological findings, and surgical management. The demographic data included gender and age, while the clinicopathologic data included tumor size, mitotic figure count, results based on the National Institutes of Health (NIH) consensus classification system, and immunohistochemistry test results, including CD117, CD34, discovered on GIST-1 (DOG1), desmin, smooth muscle actin (SMA), and S-100. The surgical management data included lesion location, surgical method, operation duration, and postoperative hospital stay.

### Single-Balloon Enteroscopy

All SBE procedures were conducted using an SIF-Q260 video enteroscope (Olympus, Tokyo, Japan) and were performed by an endoscopist. Intravenous midazolam and propofol were administered as premedication. The route for endoscope insertion, i.e., transoral or transanal, depended on the clinical signs or lesion location, as assessed via abdominal CT scanning. When the transoral route was adopted, bowel preparation was not required. When the transanal route was adopted, the bowel was prepared using a polyethylene glycol or sodium phosphate solution. If no lesion was found via 1 route, the procedure would be performed again through the other route.

### Immunohistochemistry Tests

After being fixed in paraffin, tissue samples were cut into 5-µm-thick paraffin slices. To stop endogenous peroxidase activity after dewaxing, the slices were submerged in 3% hydrogen peroxide for 10 minutes. Following this, portions were heated in a microwave for antigen retrieval and submerged in citric acid (pH 6.0). Slide-mounted sections were chilled at room temperature and treated with goat serum for 1 hour to prevent nonspecific binding. Hematoxylin–eosin staining was then applied for 10 minutes, or primary antibody incubation was performed overnight at 4°C. Finally, images were captured using a dissecting microscope (Leica DM4000), and sections were stained using a horseradish peroxidase (HRP)-3,3’-diaminobenzidine (DAB) detection kit (Beijing Biosynthesis Biotechnology Co., Ltd., Beijing, China).

### Statistical Analysis

Mono-factor analyses were performed using different methods according to the data type. The count data were expressed as frequencies (percentages), and a chi-square test was used for inter-group comparisons of these data. The continuous variables, expressed as mean ± SD, were analyzed using unpaired *t*-tests or Wilcoxon rank-sum tests. A *P*-value of <.05 was considered statistically significant. All statistical analyses were run using Statistical Package for the Social Sciences Statistics 19.0 software (IBM Corp., Armonk, NY, USA).

## Results

### Basic Patient Information

Fifty-five patients with surgically confirmed small-bowel GISTs were hospitalized at our center during the study period. Among them, 8 were excluded due to an incomplete medical history and 1 was excluded due to a malignant tumor. The remaining 46 patients constituted the study group. The study population had a mean age of 52 (range = 23-80), with roughly 89.1% (41) being under 50 years old, while half of the patients were female. The clinical presentations were melena in 35 patients and hematochezia in 11 patients. The mean duration of the symptoms was 48 hours. The hemoglobin levels ranged from 34 to 143 g/L, with a mean of 54.2 g/L. The preoperative CT scans were highly indicative of GISTs in 18 patients, revealed space-occupying lesions with an indeterminate diagnosis in 21 patients, and were normal in 5 patients following oral contrast administration. Two patients did not have a CT scan prior to surgery. Among the 39 patients with definite lesions identified via presurgery CT scans, 3 had lesions in the duodenum, 20 had lesions in the proximal jejunum, 7 had lesions in the distal jejunum, 2 had lesions in the proximal ileum, 2 had lesions in the middle ileum, and 5 had lesions in the distal ileum. Out of the 46 patients in the study group, 27 underwent SBE. The preoperative CT findings for these 27 patients were highly indicative of GISTs in 8 patients, revealed space-occupying lesions with an undetermined diagnosis in 13 and were normal in 4. Two of these patients did not undergo a preoperative CT scan.

### Enteroscopy Findings

**Figure 1.**
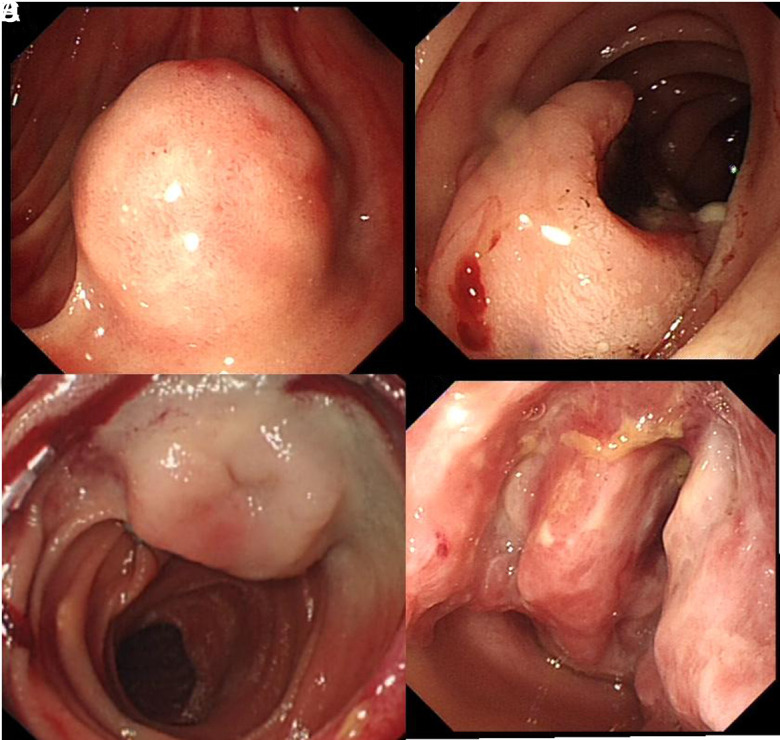
Representative enteroscopic images. (A) submucosal protuberant lesion; (B) surface congested lesion; (C) surface ulcerative lesion; (D) ulcerative infiltrative lesion.

The SBE method was performed on 27 patients. Here, only the transoral route was used for 21 of the patients, the transanal route for 2, and both for 4. The average SBE time was 91 minutes, with a range of 41-131 minutes. In 25 of the SBE patients, GISTs could be observed; however, in the remaining 2 individuals, the lesions were not visible. One of these 2 missing lesions had a diameter of 1.5 cm and was located in the mid-jejunum, while the second lesion had a diameter of 1.6 cm and was also located in this area. The endoscopic tumor characteristics can be categorized as follows: submucosal round (n = 13), submucosal sessile (n = 8), and invasive/penetrating (n = 4). Regarding the morphology of the lesions, 20 patients exhibited submucosal protuberant lesions with surface ulceration, vascular nodules/congestion, or erosion, while 5 patients had ulcerative infiltrative lesions (Figure 1A–D). Among the 25 patients diagnosed with GISTs based on SBE, 9 presented with suspicious bleeding manifestations. Endoscopic hemostasis was performed for 6 of these patients, and successful hemostasis was achieved. The remaining 3 patients exhibited no active bleeding and did not require additional treatment. Among the patients undergoing SBE, lesions were observed in the duodenum in 2, in the jejunum in 18, in the ileum in 4, and close to the junction of the jejunum in 1. Six patients who received enteroscopic biopsies underwent SBE hemostasis. Titanium clips were placed during the SBE for 24 patients.

### Surgical Findings

The surgical methods were laparoscopy for 17 (36.96%) patients, laparotomy for 27 (58.59%) patients, and conversion from laparoscopy to laparotomy for 2 (4.35%). Four patients (8.70%) had lesions in the duodenum, 32 (69.56%) had lesions in the jejunum, 8 (17.39%) had lesions in the ileum, and 2 (4.35%) had lesions around the junction of the jejunum and ileum. The average postoperative hospital stay among the patients was 8 days, with the procedure lasting, on average, 60 minutes.

### Pathological Findings

**Figure 2.**
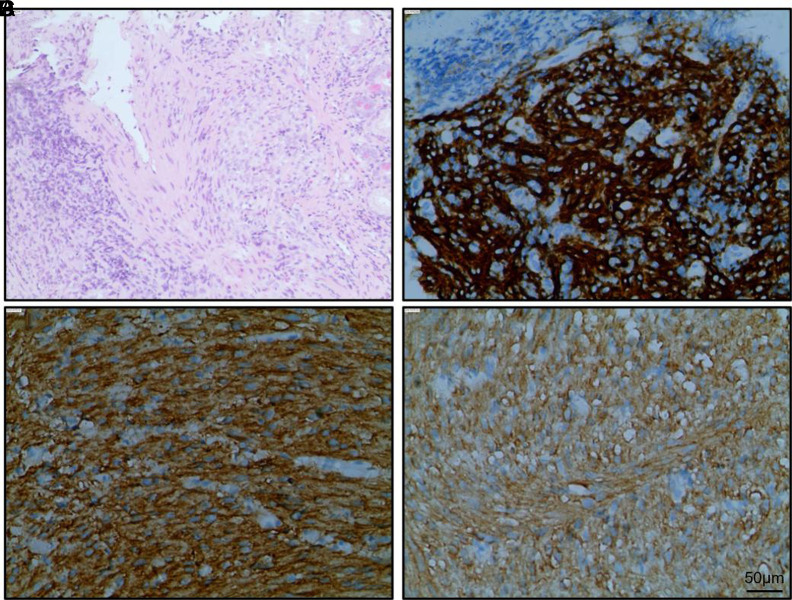
Representative hematoxylin and eosin and immunohistochemistry stained images in pathological examinations. (A) a HE staining examination; (B) IHC-stained CD117 protein; (C) IHC-stained DOG-1 protein; (D) IHC-stained CD117 protein. HE, hematoxylin and eosin stain; IHC, immunohistochemistry stain.

The mean diameter of the lesions was 5.68 ± 3.20 cm (range = 1.5-14 cm). According to the findings of the immunohistochemical test, CD117 was positive in all of the 46 patients, CD34 was positive in 28, partially positive in 8, and negative in 10, while DOG1 was positive in 43 patients, partially positive in 1, and negative in 2 (Figure 2B–D). Desmin exhibited positive results in 1 patient and partially positive results in another, with negative results among the remaining patients, while SMA exhibited positive results in 1 patient and partially positive results in 9, with negative results among the remainder (data not shown). The risk stratification for malignancy according to the NIH–Fletcher criteria for GIST risk assessment was low risk in 21 (45.65%) patients, medium risk in 4 (8.70%), and high risk in 21 (45.65%). None of the patients were “extremely low risk.””

### Correlation Between the Endoscopic Characteristics and Symptoms and Histology

The SBE method can provide valuable information on the endoscopic characteristics of GISTs, including tumor location, diameter, shape, surface ulcers, and bleeding. In accordance with the NIH–Fletcher GIST risk assessment guidelines, the risk of GIST malignancy was categorized into 4 groups: extremely low risk, low risk, medium risk, and high risk. To explore the factors associated with GIST risk, multiple logistic regression analysis was conducted using age, sex, tumor location, diameter, shape, and blood infiltration as the independent variables. The results revealed that the invasive/penetrating characteristics of GIST under SBE evaluation are significantly correlated with the risk level of GIST malignancy (*P* < .05) (Table 1).

## Discussion

The most prevalent stromal tumors in the gastrointestinal tract are GISTs, which develop from Cajal’s interstitial cells.^9^ A GIST may be brought on by oncogenic mutations in the CD117 (KIT) or platelet-derived growth factor receptor alpha (PDGFRα) genes.^10^ Over 95% of GISTs express the c-KIT proto-oncogene, CD117, and 70%-90% of GISTs express the human hematopoietic progenitor cell marker, CD34. Patients with GISTs also have mutations in the exon 18 of the PDGFRα in addition to c-KIT. Exons 9 and 11 were also shown to have c-KIT mutations.^11,12^ In this study, the sample consisted of an equal number of male and female patients, and 89.13% of the patients were aged 50 or older. The percentages of patients with positive CD117, CD34, and DOG1 expression were 100.00%, 78.26%, and 95.65%, respectively.

The majority of GISTs are asymptomatic and are only discovered by chance during surgeries for other reasons or during abdominal imaging. Patients with symptoms often present with gastrointestinal bleeding, obstructive symptoms, abdominal pain, and/or weight loss, among other symptoms. According to several studies,^13,14^ small-intestine GISTs have a poorer prognosis than gastric GISTs, and patients with the former are more likely to experience gastrointestinal hemorrhage than those with the latter. Therefore, attention should be paid to small intestinal GISTs with gastrointestinal bleeding as the main manifestation.

The major techniques for diagnosing small-intestine illnesses include abdominal CT scanning, capsule endoscopy, and balloon-assisted endoscopy, while each has a modest but real risk of misdiagnosis.^4,15^ The idea of deep enteroscopy was first proposed when Yamamoto et al^16^ developed DBE in 2003. Following this, spiral enteroscopy and SBE were also made available.^17^ Previous studies^18^ have demonstrated the preoperative diagnostic utility of SBE for the effective surgical treatment of 3 instances of gastrointestinal cancers of separate etiologies. Small-bowel illness is more frequently discovered via SBE.^19,20^ In the present study, 27 patients with small-bowel GISTs underwent SBE examination, and lesions were clearly detected in 25 cases, with a detection rate of 92.59%. It is evident that SBE has a positive application impact in the diagnosis of patients with small-bowel GISTs.

The endoscopic characteristics of SBE in small-intestine GISTs have not been systematically evaluated. Yoo et al^4^ demonstrated that, among 7 patients with small intestinal GISTs, 4 had undergone DBE and exhibited submucosal protuberant lesions, while 3 had undergone capsule endoscopy, with 2 exhibiting submucosal protuberances and 1 ulcerative infiltration. In the present study, the endoscopic morphology of small-intestine GISTs under SBE testing was assessed. Small intestinal GISTs can be submucosal, round, sessile, or invasive/penetrating. Martinez-Alcalá et al^21^ reported that the small intestinal GISTs examined via DBE also exhibited submucosal roundness, submucosal baselessness, and invasiveness, similar findings to our own.

Due to the limited diagnostic evidence provided by biopsy specimens, endoscopists often refrain from performing biopsies on most lesions. While small intestinal biopsy is considered the gold standard technique for identifying small-bowel disorders, it is not routinely recommended for disease monitoring, especially among asymptomatic patients, due to its invasive nature and the associated risks and costs.^22^ According to Dolu et al’s^7^ findings, all patients (100%) whose histological diagnosis via DBE was a benign small-bowel tumor had endoscopically suspected polyps (n = 48). However, in the present study, some of the lesions resembled lipomas due to their round, submucosal form and yellowish core. Only our prior experience with small intestinal GISTs led us to insist on surgical exploration and treatment for these individuals since none of these lesions could be identified as GISTs using a biopsy.^21^ In addition, biopsies are often traumatic, increasing the risk of infection and needle tract dissemination. In the present study, endoscopic biopsy was performed in 6 patients, with only 2 of them having a definite diagnosis. Nakano et al^23^ also found that antegrade DBE was the preferred method for investigating small-bowel GISTs, while the diagnostic utility of a biopsy examination was limited.

This study has a number of limitations. First, this was a single-center retrospective study with a small sample size, meaning there was the possibility of selection bias. Second, the relatively low incidence of small intestinal GISTs led to the long duration of this study. With the recent changes in surgical techniques, instrumentation, and post-surgical management strategies, the extended selection period might have resulted in a greater bias among the participants. Multi-center studies with a larger patient population and a prospective design are clearly needed to validate our observations and assess their prognostic relevance.

The endoscopic characteristics of small-intestine GISTs under preoperative SBE evaluation included submucosal round, submucosal sessile, and invasive/penetrating. The invasive/penetrating characteristics of GIST under SBE evaluation are significantly correlated with the risk level of GIST malignancy.

## Figures and Tables

**Table 1.** Results of Multiple Logistic Regression Analysis

	*B*	Standard Error	Wald	*P*	95% CI
Constant (*y* = 1)	−2.225	3.937	0.320	.572	−9.942-5.491
Constant (*y* = 2)	−1.293	3.913	0.109	.741	−8.963-6.377
Gender	2.186	1.399	2.444	.118	−0.555-4.928
Age	−0.045	0.063	0.502	.479	−0.168-0.079
Hemoglobin	−0.015	0.035	0.191	.662	−0.084-0.053
Shape	3.367	1.610	4.373	.037	0.211-6.522
Hemostasis	1.416	1.389	1.040	.308	−1.306-4.137
